# Correction: Infant-directed input and literacy effects on phonological processing: Non-word repetition scores among the Tsimane’

**DOI:** 10.1371/journal.pone.0274528

**Published:** 2022-09-08

**Authors:** Alejandrina Cristia, Gianmatteo Farabolini, Camila Scaff, Naomi Havron, Jonathan Stieglitz

Fig 1 is incorrect. The authors have provided a corrected version here.

**Fig 1:**
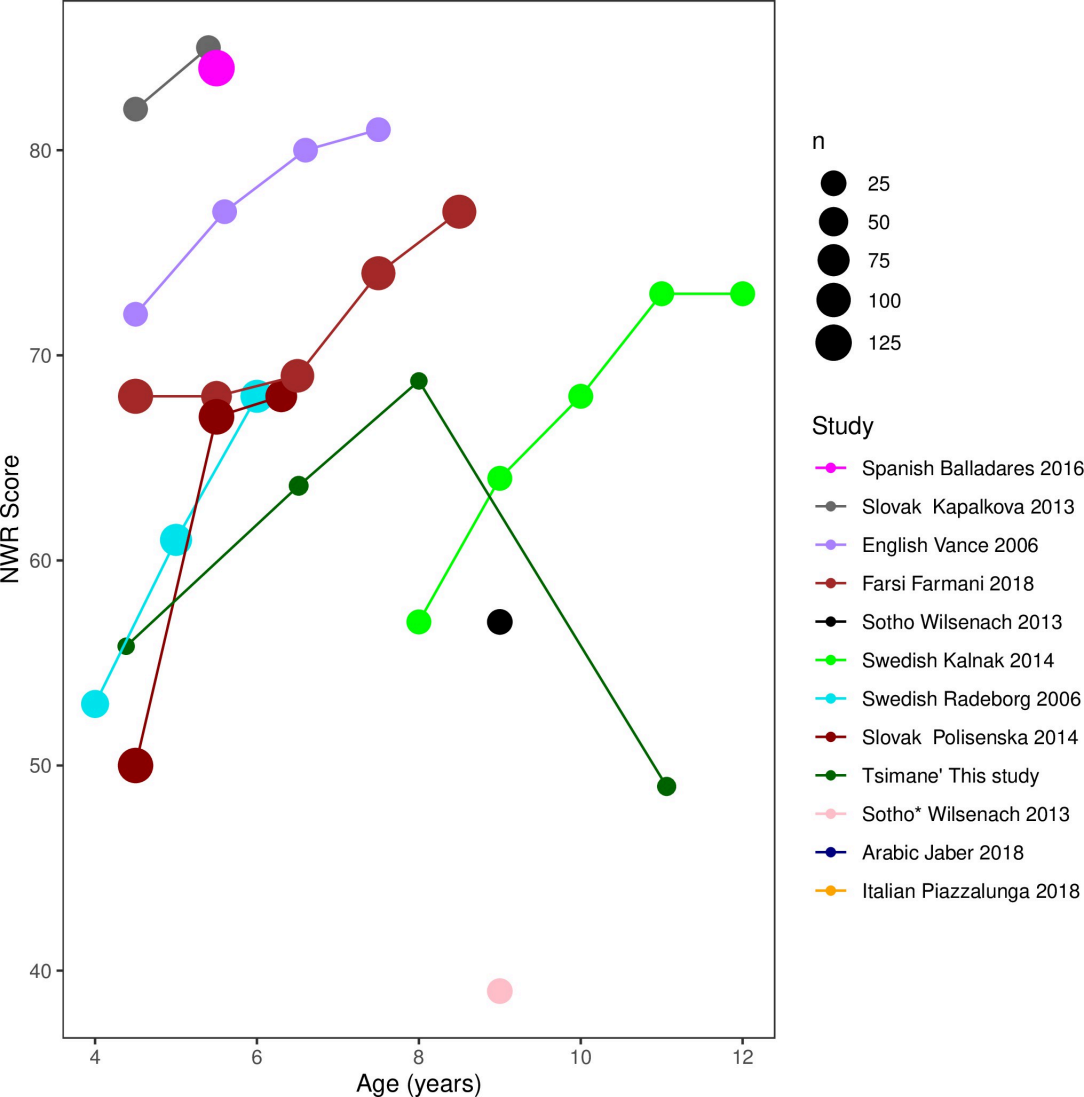
NWR scores as a function of age (in years), study (first author and year regardless of the number of authors). Study legend is sorted from highest to lowest average to facilitate linking. The size of the circle indicates sample size.
