# Supplementary material for: Infant-directed input and literacy effects on phonological processing: Non-word repetition scores among the Tsimane’
Source: PLoS One. 2020 Sep 11;15(9):e0237702. doi: 10.1371/journal.pone.0237702 (PMC7485875; doi:10.1371/journal.pone.0237702)
Supplement: Attachment — Submitted filename: nwr-tsi PONE-D-19-31695 Response to Reviewers.pdf [file pone.0237702.s001.pdf]

Dear Editor,

We would like to thank you and the two reviewers for your extremely thoughtful and useful comments. We were thrilled to get such thorough feedback from experts on this study. The manuscript has clearly been strengthened as a result of incorporating these constructive comments.

We include below a point-by-point reply to your and the reviewers' comments. In a nutshell, we:

- Added text in the Introduction (lines 239-288, *all line/page numbers refer to the unmarked version - not the tracked-changes version*), providing further information on the Tsimane' language.
- Added text in the Preprocessing subsection (lines 516-528), with further analyses of the ways in which error patterns likely reflect phonological processing.
- Added text in the Discussion (lines 743-831), reflecting on whether relatively lower NWR scores reflect phonological processing affected by literacy and/or early input, or just discomfort with the task.
- Added all citations recommended by reviewers or yourself.
- Toned down claims of causality and announced low power from the outset.

In addition, we have made changes throughout the manuscript to facilitate reading, to comply with the journal's formatting requirements, and to be more accurate in our terminology. We have also revised the author order after more extensive study of contributions.

We look forward to hearing about your and the reviewers' reaction to this version.

Yours,

Alejandrina Cristia, on behalf of all authors

PONE-D-19-31695

Infant-directed input and literacy effects on phonology: Non-word repetition accuracy among the Tsimane'

PLOS ONE

Dear Dr. Cristia,

Thank you for submitting your manuscript to PLOS ONE. After careful consideration, we feel that it has merit but does not fully meet PLOS ONE's publication criteria as it currently stands. Therefore, we invite you to submit a revised version of the manuscript that addresses the points raised during the review process.

Both reviewers note that the paper has value and appeal to the field, particularly in investigating a non-WEIRD population that is extremely underrepresented in the literature. At the same time, they highlight several significant issues with the framing of the paper, breadth of literature covered, and interpretations drawn. I agree that these issues should be addressed to make the submission suitable for publication. I have summarized the main points made by each reviewer, with their specific comments below.

Both reviewers agree that there are gaps in the literature covered and recommend incorporating additional literature, as well as expanding upon some of the studies that are already mentioned. Specifically, Reviewer 1 asks **for more coverage of the research relating to literacy and the development of phonological knowledge, and discussion of the quality (rather than just quantity) of children's language input on language development.** Reviewer 2 asks for **more information about the Tsimane language (particularly regarding the phonological inventory and allophonic variation), amount of child language input across different communities, results from additional nonword repetition tasks, and discussion of other research that has tested phonological awareness in remote communities (e.g., Sharma, Wigglesworth, Savage & Demuth, 2020).**

We have added these references and this information, and we expand on each point below.

While you do explicitly acknowledge that your analyses are exploratory, **Reviewer 1 advises that this should be addressed from the outset of the paper. Reviewer 2 expresses concern that the interpretations are still overextended given that it is not yet established whether the NWR task here reliably measures phonological skills, and given the confounds in the task and population within the experiment and relative to prior literature. This is particularly with regard to the question of amount of early language input.**

We now mention from the outset that our analyses are exploratory, and have toned down any claims for causality. Additionally, we directly point out why we favor our interpretation over alternatives while remaining clear on the absence of proof for causality in the new section in the discussion (lines 743-831). We respond to all reviewer comments in more detail below.

Still, as Reviewer 2 points out, this paper reflects the challenge of adapting typically lab-based methods to the field in a remote setting, and in that has significant value to others looking to test in underrepresented populations. As such, Reviewer 2 recommends lending more discussion to the process/challenges that were certainly faced in such an endeavor to help future researcher anticipate and accommodate similar challenges.

We agree that this would be a valuable service to science and have added a supplementary material document to address it.

We would appreciate receiving your revised manuscript by Apr 09 2020 11:59PM. When you are ready to submit your revision, log on to https://www.editorialmanager.com/pone/ and select the 'Submissions Needing Revision' folder to locate your manuscript file.

If you would like to make changes to your financial disclosure, please include your updated statement in your cover letter.

To enhance the reproducibility of your results, we recommend that if applicable you deposit your laboratory protocols in protocols.io, where a protocol can be assigned its own identifier (DOI) such that it can be cited independently in the future. For instructions see: http://journals.plos.org/plosone/s/submission-guidelines#loc-laboratory-protocols

Please include the following items when submitting your revised manuscript:

- **A rebuttal letter that responds to each point raised by the academic editor and reviewer(s). This letter should be uploaded as separate file and labeled 'Response to Reviewers'.**
- **A marked-up copy of your manuscript that highlights changes made to the original version. This file should be uploaded as separate file and labeled 'Revised Manuscript with Track Changes'.**
- **An unmarked version of your revised paper without tracked changes. This file should be uploaded as separate file and labeled 'Manuscript'.**

Please note while forming your response, if your article is accepted, you may have the opportunity to make the peer review history publicly available. The record will include editor decision letters (with reviews) and your responses to reviewer comments. If eligible, we will contact you to opt in or out.

We look forward to receiving your revised manuscript.

Kind regards,

Karen E. Mulak, Ph.D.

Academic Editor

PLOS ONE

Journal Requirements:

When submitting your revision, we need you to address these additional requirements.

1. **Please ensure that your manuscript meets PLOS ONE's style requirements, including those for file naming.** The PLOS ONE style templates can be found at

http://www.journals.plos.org/plosone/s/file?id=wjVg/PLOSOne_formatting_sample_main_body.pdf and http://www.journals.plos.org/plosone/s/file?id=ba62/PLOSOne_formatting_sample_title_authors_affiliations.pdf

We have complied with this request

2. **Please provide additional details regarding participant consent. In the Methods section, please state how verbal consent was recorded and whether the ethics committee approved this consent procedure. We note that consent was obtained from the parents of the minors that took part in the study. Please clarify whether provided explicit permission for their children to take part in research.**

The Participants section now starts:

> ***"Institutional IRB approval was granted by University of New Mexico (HRRC # 17–262), as was informed consent at three levels: (1) Tsimane' government that oversees research projects (Gran Consejo Tsimane), (2) village leadership and (3) study participants. We made a public presentation at each village where we explained the general goal of the research, to study language acquisition, and demonstrated some of the methods. After this, people visited our camp and/or we visited them in the context of other studies. At this time, they were asked whether they wanted to additionally participate in this study, which took between 1.5 and 6.5 minutes. In addition, participants' reactions were monitored to ensure they participated in their own terms (e.g., participants could stop repeating the items). Participants were not compensated for participating in the NWR game specifically, and***

thus there was no gift to promote higher NWR scores. Instead, participants were compensated in lump sum for the battery of protocols they participated in. Participants consented verbally, for themselves and for their children. This verbal consent/assent procedure was approved by the above-mentioned IRB."

3. **Please note that PLOS ONE uses a single-blind peer review procedure. We would therefore be grateful if you could include in the information that has been anonymised for peer review in the manuscript.**

This information has now been included.

[Note: HTML markup is below. Please do not edit.]

Reviewers' comments:

Reviewer's Responses to Questions

Comments to the Author

1. Is the manuscript technically sound, and do the data support the conclusions?

The manuscript must describe a technically sound piece of scientific research with data that supports the conclusions. Experiments must have been conducted rigorously, with appropriate controls, replication, and sample sizes. The conclusions must be drawn appropriately based on the data presented.

Reviewer #1: Partly

Reviewer #2: Partly

---

2. Has the statistical analysis been performed appropriately and rigorously?

Reviewer #1: Yes

Reviewer #2: I Don't Know

---

3. Have the authors made all data underlying the findings in their manuscript fully available?

The PLOS Data policy requires authors to make all data underlying the findings described in their manuscript fully available without restriction, with rare exception (please refer to the Data Availability Statement in the manuscript PDF file). The data should be provided as part of the manuscript or its supporting information, or deposited to a public repository. For example, in addition to summary statistics, the data points behind means, medians and variance measures should be available. If there are restrictions on publicly sharing data—e.g. participant privacy or use of data from a third party—those must be specified.

Reviewer #1: Yes

Reviewer #2: Yes

---

4. Is the manuscript presented in an intelligible fashion and written in standard English?

PLOS ONE does not copyedit accepted manuscripts, so the language in submitted articles must be clear, correct, and unambiguous. Any typographical or grammatical errors should be corrected at revision, so please note any specific errors here.

Reviewer #1: Yes

Reviewer #2: Yes

---

5. Review Comments to the Author

Please use the space provided to explain your answers to the questions above. You may also include additional comments for the author, including concerns about dual publication, research ethics, or publication ethics. (Please upload your review as an attachment if it exceeds 20,000 characters)

Reviewer #1: The basic idea of the paper is fantastic, and is important, so I would definitively like it to be published.
Thank you!

However, it suffers from several limitations.

The first and more important one is that it is exploratory, and based on very small samples. Yet, except for a rapid note in the method (line 231, p. 6, "All analyses must thus be considered as exploratory), we must wait until line 527, in the discussion, to see that the Authors acknowledge that "This paper reports on exploratory analyses ….". This should be acknowledged much earlier in the paper, already in the Introduction. The idea the Authors explore and the challenge this represents makes it perfectly acceptable to present an exploratory paper.
We have added:

- **the word "preliminary" to the first paragraph of the article**

- "*As discussed in subsequent sections, the sample size is fairly small and analyses were not pre-registered, and should be considered exploratory.*" **in the first paragraph under the "The present study"**

Also, it is surprising that reading ability of the participants was not tested, but self-reported. This limitation should also be clearly acknowledged.

We have added:

- **"self-reported" in "we compare […] self-reported readers and non-readers to assess the potential impact of literacy" in "The current study" section, and in several other places**
- **"it is possible that there is more noise than in previous studies in both our children's and adults' data because reading proficiency and schooling were self-reported rather than directly measured" in the Discussion, under "The effects of literacy and/or education"**

Second, there are some important details and references missing as regards the effects of literacy on nonword repetition. For instance, in discussing the effects of literacy vs. schooling on memory, the authors should mention that there is at least one longitudinal study reporting a small but significant improvement in phonological memory (nonword repetition, with NO mixing with real words) on seven Portuguese adults who were unschooled and fully illiterate at the beginning of the study, but were successfully taught to decode in 3 months (Kolinsky et al. 2018, Reading & Writing). This improvement in nonword repetition was correlated positively with progress in phoneme awareness, as assessed by a phoneme deletion task. Also note that performance on nonword repetition at the beginning of study (i.e., when participants were still fully illiterate, as it was the case in the two pre-tests, T1 and T2) was very low. Interestingly, though, their performance seems higher than the one observed in the present study, at least when items of similar length are considered:65,62% on average on items with 1 to 4 syllables, 62,5% on average on disyllabic items.

We have added this reference on page 13 in the literature review section of the introduction, and also made a more exhaustive search among papers authored by Morais and papers citing this 2018 study. We agree with your reading that performance in their study is higher than in ours for both 1-2 and 3-4 syllable-long items, so we have minimally altered these sentences from the discussion:

> *"We predicted that Tsimane' NWR scores would be higher than that observed in previous studies because we framed the exercise as an enjoyable game, rather than a scientific data collection task. However, we find descriptively lower levels of NWR scores when comparing Tsimane' adults with illiterate participants studied in previous work, even when focusing on relatively simple non-word items. As just discussed, overall lower levels of NWR scores in this group than others previously studied could relate to lower levels of infant-directed input for our Tsimane' adult participants when they were children (compared to non-readers and illiterates who have participated in previous work, although we know of no quantitative evaluation of infant-directed speech levels in these other populations)."*

As regards the effect of item length, line 548 p. 13 it is said that “It is infrequent for studies to report performance separately as a function of item length”. We agree with that, and this is why it would be important to compare the data of the present study with those observed by Kolinsky et al. (2018) in fully illiterate Portuguese adults (see their Appendix): as I already reported, at pretest their participants presented 65,62% on average on items with 1 to 4 syllables, 62,5% on average on disyllabic items.

We have reorganized this paragraph, included a reference to Kolinsky, and added a Figure 8 to more easily integrate our results with those of others:

> ***"First, one may wonder whether the items we used were phonologically more complex than those employed in previous work. It is infrequent for studies to report NWR scores separately as a function of item length. Nonetheless, we found some studies that did so [@de2004degree; @piazzalunga2019articulatory; @kolinsky2018completely], and combined them with studies that used non-words that were either 1-2 or 3-4 syllables in length [@castro1998illiterate; @jabere2018xperiment;@Tsegaye2011]. We also split up NWR scores by age, literacy (among adults), and non-word length in our own data. Results are represented in Fig 8, which shows that Tsimane' participants' NWR scores were lower than those of participants in previous work (matching for age, literacy, and non-word length)."***

Also, presentation of the data is unsatisfactory as regards the effect of item length. Indeed, average group values for each nonword length are not presented (only individual scores in Figure 4), and, no separate scores are provided for the “readers” vs. “non-readers”. This would be very interesting, as there is no reason to expect a huge (readers vs non-readders) group difference on short (mono or disyllabic) items, but such a group difference is expected on longer items (perhaps already 3-syllables long; probably 4-syllabes long items).

We have coded differently readers versus non-readers in this Figure and all others. About this figure specifically, please note that there do not seem to be greater differences between the groups for longer items.

In any case, in the abstract, the formulation “we found weak evidence that literacy and/or education improves performance in non-word repetition” is ambiguous; please reformulate. On lines 648-650 (p. 15), it is much more exactly said that “(..), we found some evidence that literacy and/or education improves performance in non-word repetition, although these results should be interpreted with caution given the small sample size”.

The abstract now reads:

> **"Additionally, we found some evidence that literacy and/or education improves performance in non-word repetition, although results should be interpreted with caution given the small sample size."**

Finally, the authors should not restrict the discussion to the quantity of verbal input provided to children, but include the quality of that input (see discussion in Golinkoff et al., Child Development 2019, as regards quality speech directed to children rather than overheard speech).

We have added the following text in the discussion:

> **"It would also be interesting for future work to incorporate quality metrics (e.g., lexical diversity, @rowe2012longitudinal) and extend the study of effects from input quantity to input quality, although we are less certain of how this can be properly evaluated across very diverse languages."**

We respectfully submit that any further discussion goes beyond the scope of our paper. The motivation to look at NWR in this population came from studies of quantity, not of quality. We understand the reviewer is interested in quality, and we agree that it is of broad interest and theoretically significant, but we believe it is beyond the scope of the present paper to extend that discussion.

Minors and typos
Table 1: presentation is not clear, in particular as whether schooling was controlled for (e.g., all the "literate" participants in Castro-Caldas et al 1998 had attended school) . More generally, the terminology adopted in that table as well as on p. 4 (bottom) is confusing: the Authors speak about "illiterate", "non-readers" and "non-literates". What is the difference ?(I suspect none).

We have added to the table caption "In Group, we use the terminology of each paper to identify each of the tested group (see footnote 1 for details)." This is important, in our view, to allow readers to check that we have summarized the sources appropriately. In the main text, we also added this footnote

> **"@Reis1997 classified as illiterate only participants who met several conditions including not reading any kind of written material (except for their names in some cases) and being born of illiterate parents; in contrast, literates had acceptable performance on tests of reading and writing. @castro1998illiterate tests a subset of participants of @Reis1997 and further adds that functional illiterates (with exposure to reading and writing, likely through schooling) were excluded. In @de2004degree, non-readers and readers both self-identified as illiterates; among these, non-readers were participants who did not read at all, whereas readers could read at least one word. Controls, in contrast, attended at least 5 years of school. Illiterates in @kosmidis2006lexical had never attended school and although they could sign their initials, they failed in a reading test consisting of identifying graphemes and reading a paragraph. Literates in this study not only had attended school (1-9 years) but also reported reading regularly. Illiterate participants in @Tsegaye2011 could not read or write a test including letters and simple words, whereas literate participants had 7-10 years of schooling. Finally, @kolinsky2018completely's study will be introduced below as having a pre-post design on the same participants, who before training had attended school irregularly 0-2 years."**

Line 61 p. 3: correct "enviroments" : "environments"
Corrected.

"Castro" should be replaced by "Castro-Caldas" in table 1 (=his last name)
Corrected.

Replace "Louriero" by "Loureiro" in table 1
Corrected.

Loureiro et al 2004 is missing in the Reference list
Corrected.

In table 1 the performance for Castro-Caldas 1998 is not for long nonwords, but average performance
Since non-words were 3-syllables long, they can be classified as "long" according to our criteria (3-4 syllables). We now mention these numbers both in "overall" and "long" (except that now the table does not have both overall and short/long, but the scores for long are represented in Fig 8).

Line 424 on p. 10: “paraticipants” should be replaced by “participants”
Corrected.

Line 519, p. 12 “aduls” should be changed into “adults”
Corrected.
We thank you for the precise reading of our paper, and apologize profusely for these typos.

Reviewer #2: This study about language experience in the Tsimane community and its role in phonological representations. While it is an interesting project, and important in terms of addressing child language in a community which offers a different perspective, there is certainly work required on the interpretation in this study. I would not want to see this published without some rethinking on what the results could really mean. It is not currently clear if the findings are truly about long-term effects on phonological representations, or simply task effects i.e. the actual experiment - framed as a game - is not a typical kind of activity for this community. That is OK, but the findings are currently a little opaque.

Thank you for your general feedback and for expressing healthy skepticism about our task validity. Some of these issues raised were on our own minds as well, given that we adapted the task design to maximize ecological validity. We now better acknowledge these shortcomings.

Your main concern is now addressed in a new section in the Discussion ("Effects of input, literacy, or task oddity?"), where we raise the general point alluded to here: that the problem might be that our task and/or the general non-word repetition idea is not good to pick up on phonological skills. In it we discuss:

- **are our findings due to the fact that our measure is a game, versus a task that is presented as such (as it has been done in previous research)?**
- **Is it because these are non-words, and non-words are by their very nature difficult for illiterates and/or for cultures like the Tsimane'?**
- **Does the experiment highlight people's real abilities?**

It is not currently clear if the experiment is useful for highlighting people's real abilities.

As mentioned above, this is addressed in a new section in the Discussion ("Effects of input, literacy, or task oddity?"), where we raise the general point alluded to here: that the problem is that our task and/or the general non-word repetition idea is not good to pick up on phonological skills.

Also, some languages allow a lot of variation in phonetic realisations, so how can we be certain that "errors" are truly "errors" and whether the results are truly shining a light on phonological representation? I feel more evidence or justification is required.
At the end of the Preprocessing subsection of the Results, we added more details that many shed some light on this. First, we added before the figures showing performance as a function of item length and complexity that these figures clearly show phonologically-expected effects. Second, we added a whole paragraph reading as follows:

> **"Third, one could argue that people diverged from the model because perhaps in the participants' language mispronunciations are tolerated. It is indeed the case that some languages allow a great deal of allophony. Although there is unfortunately little research on the phonetics and phonology of the Tsimane' language, our own experience in the field is that it is not the case that there is rampant allophony and great mispronunciation tolerance among the Tsimane'. Languages allowing a great deal of allophony often have simple inventories, e.g. 3-5 vowels, but Tsimane' has a fairly complex inventory including widespread contrastive vowel nasalization, the presence of two series of central vowels, and the presence of a 4-way lingual (dental versus alveolar versus post-alveolar versus palatal) contrast. Moreover, participants sometimes attempted to repeat an item they had not repeated accurately, showing that they were aware of the deviation."**

I list my specific comments below:
In the introductory sections it would be very useful to understand the specifics of the Tsimane language - where exactly is it spoken (a map would be very helpful), how many speakers, what language family, and what is the phonological inventory (especially important given the topic). Also just a mention of the different spellings of the language name would be useful.
We have added a section introducing the Tsimane' language and community. We have not added a map because we feel several good ones are present in previous literature (and it would delay publication to ask for permission to reuse them). Instead, we refer readers to the following one:

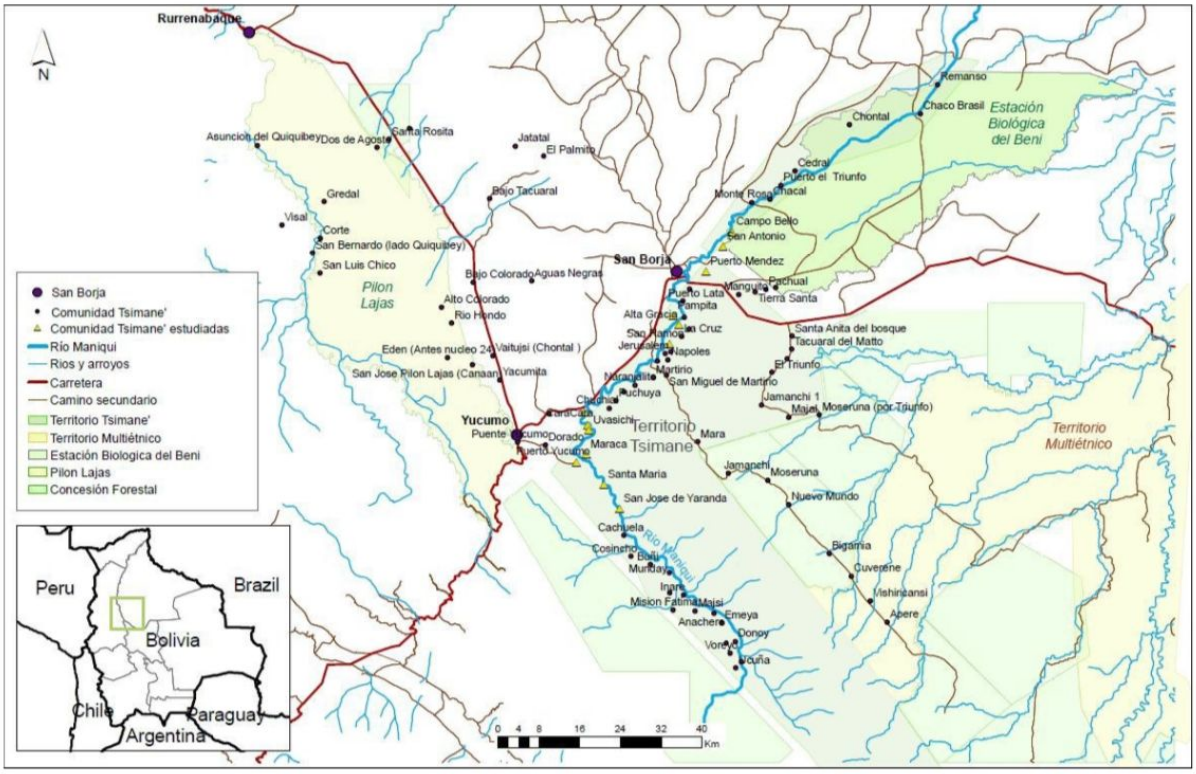

p.2 line 60 onward - here I was expecting a nod to research which has already addressed this topic, such as work by Katherine Demuth.

Page 2 line 60 onward read "if phonological development is driven by lexical development, and lexical development is driven by infant- and child-directed input, then environments that are associated with little infant- and child-directed input should also have less robust or later-developing phonological representations." We are familiar with the work of Katherine Demuth, but since we were uncertain about what the reviewer was alluding to, we checked again. The Editor highlighted a paper that came out in January 2020, on the correlation between auditory processing and phonemic awareness. This paper does not seem like a good fit for this section of the manuscript, so now we cite it in the Discussion:

> ***"All of that said, we do not want to leave the reader with the impression that we have proved causality. Lower scores in one task in one population could be due to a host of reasons. For example, in a recent paper on perceptual skills among school-aged children aboriginal to Australia, it was found that children scored relatively poorly in phonological awareness, and individual variation in these scores correlated with individual variation in psychoacoustic tests [@sharma2020binaural]. We have not tested hearing among the Tsimane', and it is possible that a higher proportion of Tsimane' have hearing difficulties than other populations represented in the literature, and that variation in auditory skills would impact their non-word repetition NWR scores. This is then a potential confound that would need to be addressed in future work."***

Looking more generally, we could not find anything in Demuth's work where she states that phonological representations are less robust or develop later when there is little child-directed speech. We thought the reviewer may be thinking of Song et al.

(2018), which is one among many studies investigating the correlation between child-directed speech and vocabulary. We therefore added this paper to the citations in this phrase "individual and sociocultural variation in lexical development is at least partially explained by the quantity and quality of the interaction the child is involved in".

p.2 line 37 - technically, children are exposed to language before birth, in utero. I take your point that children are not "pre-programmed" in general, but I would reword this a little.
We have rewritten these sentences. They now read "Instead, each child must learn the phonological inventory of her language(s) from her language-specific experiences (including those in utero)."

p.3 line 55 - the non-word "beng" should have italics or stand out from the main text, it looks a little like a typo currently.
Corrected.

p.3 line 97 - I am not sure why "anonymized" is not just listed the author's name? The document I have is not anonymized otherwise.
We have replaced all "Anonymized".

p.3 line 99 onward. I think the approach of doing a google scholar search in this way to understand past literature is not fully appropriate. I think a proper / comprehensive literature review, not excluding any papers because they were not in the top 20 "hits", would be more useful.
We agree that a systematic review would be incredibly useful for the field, but this goes beyond the scope of the present paper.

p.4 I am not convinced that the word "performance" should be used to talk about people's ability in their own languages. It seems that the language is used for day-to-day communication, so maybe "performance" is not the right word for this.
Changed performance to phonological skills.

p.4 line 158 - I think it is a really crucial point that "arbitrary tasks" are not typical for people with little education. This really needs to be explored further.
As mentioned above, we have added a new section in the Discussion ("Effects of input, literacy, or task oddity?"), where we raise the general point alluded to here: that the problem is that our task and/or the general non-word repetition idea is not good to pick up on phonological skills. We have made no changes to this line, which still reads "Notice that literate and illiterate adults typically do not only differ in their ability to read and write, but also in the fact that the former have been more extensively schooled, and thus trained in performing arbitrary tasks and tests, as discussed in @Kosmidis2011."

p.6 line 210 I take the point about the critical period, but I believe it is also true that children can sound native in another language up until around 8 - so perhaps a bit more discussion / more references here would be useful.

The precise age is not so crucial, and we have made this clearer by adding the following to our first discussion of a critical period "A similar argument can be made for any critical age threshold. For instance, imagine that the most sensitive period for phonology exposure starts closing at age 12 years. Children who have received little input from birth to 2 years of age can only catch up with children who have received more during the same period if the former receive more linguistic input than the latter during the 2-12 years period." We have also removed the age two years from line 210.

p. 6 Methods - I think the work around the methodology needs more discussion. It sounds like actually a lot of work was done in difficult circumstances, and that various changes had to be made in the course of executing the study. This would be useful for other researchers to know about.

We agree that it would be a great service to science to talk more about the difficulties and challenges that were involved in field testing. We thank you for this suggestion. We have added a supplementary materials document in which we explain the general situation of our field work, we mention the difficulties we came across and the solutions we implemented, and we list some improvements that could be done in future work.

p.6 line 235, p.7 line 240, line 243 - again "Anonymized" is not really needed here.

We have replaced all "Anonymized".

p.7 Here is where it would be useful to reflect back on the phonological inventory of the language. It would also be especially useful to know, in general, how frequent the sound patterns are - is the palatal especially common, how much phonetic variation is allowed, and was this incorporated into the design of the project? Things like this help to better understand the context.

As mentioned above, we added this paragraph:

> **"one could argue that people diverged from the model because perhaps in the participants' language mispronunciations are tolerated. It is indeed the case that some languages allow a great deal of allophony. Although there is unfortunately little research on the phonetics and phonology of the Tsimane' language, our own experience in the field is that it is not the case that there is rampant allophony and great mispronunciation tolerance among the Tsimane'. Languages allowing a great deal of allophony often have simple inventories, e.g. 3-5 vowels, but Tsimane' has a fairly complex inventory including widespread contrastive vowel nasalization, the presence of two series of central vowels, and the presence of a 4-way lingual (dental versus alveolar versus post-alveolar versus palatal) contrast. Moreover, participants sometimes attempted to repeat an item they had not repeated accurately, showing that they were aware of the deviation."**

We also moved the text regarding phonological descriptions of the language to the added section in the Introduction, on the Tsimane' language and people, which we

added in response to one of your comments. In that section also, we added a Figure portraying the phonological inventory, including frequency of each phoneme in Gill (1999)'s dictionary.

To finish addressing this comment, we have now added the following text in the Stimuli section:

> ***"We calculated word shape (defined as sequences of consonants and vowels) and phoneme frequencies using the 16,518 Tsimane' lexical entries in the Tsimane'-to-English portion of @gill1999chimane's dictionary. In terms of overall shapes, we found 614 unique types in the dictionary (with 351 that were hapaxes, having a frequency of 1). The frequencies (number of tokens for that type divided by number of tokens of all types) and ranking of the shapes (from most to least frequent) present in our stimuli were as follows. CVC ranked sixth with 3.99% of tokens having this type (in each case, we give examples of non-words in our stimuli having this shape: *sin, taf, wik*). CVCV ranked ninth with 3.04% (e.g., *dadi, bike, kito*). CVCCVC ranked first with 8.19% (e.g., *boxtim*). CVCVC ranked second with 9.24% (e.g., *fisek, potex, wodix*). VCVCV ranked 47th with 0.36% (e.g., *ajaSa, oSiso*). CVCVCV ranked eighth with 3.11% (e.g., *kijeki*). CVCCVCV ranked 10th with 2.96% (e.g., *deSpote*). CVCVCCV ranked 13th with 1.84% (e.g., *koxtika*). CVCVCVCV ranked 20th with 0.92% (e.g., *Tedikoti*). CVCVCVCVC ranked 16th with 1.57% (e.g., *tipijerax*). Thus, our stimuli sampled a wide range of frequencies, from the two most frequent word shapes (CVCCVC, CVCVC) to a shape ranked 47th (VCVCV) out of a potential 351 non-hapax shapes.***
>
> ***As for phonemes, we found 118,627 tokens of 41 phoneme types in the inventory (see Fig 2). We reasoned that the frequency and ranking of individual phonemes was less informative than the average phoneme frequency for each one of our non-words (i.e., for *sin*, the average of the frequency of /s/, /i/ and /n/). Our items' average phoneme frequency ranged from 2.57% to 7.95%, with a mean at 5.6%.***
> ***"***

Finally, we have added an analysis of performance as a function of word shape and average phoneme frequency. Neither factor was significant, but there was a trend in the expected direction for phonemes (better performance for items with higher average phoneme frequency).

p.8 line 312 - reference 49 is incomplete
We have removed this reference.

p.9 In the "scoring" section, any time a non-word is listed it should be in italics, or separated from the main text somehow for readability.
Corrected.

p.9 line 375 - I would consider replacing "refusing to make an attempt" with "not making an attempt".
Corrected.

p.9 line 376 - presumably the issue about not being able to determine a speaker's identity in your recordings is a good learning opportunity that could be described / explored a little further in the paper. What solutions could you offer other researchers who have to use a similar experimental condition? Perhaps individual lapel microphones? Obviously it is very important to be sure which data belongs to which person.

We have added a supplementary materials document in which we explain the general situation of our field work, we mention the difficulties we came across and the solutions we implemented, and we list some improvements that could be done in future work.

p.10 in terms of what affects "performance", I think we could consider phonological complexity and also frequency of certain sound patterns as potential factors. I think this should be addressed in some way.

Phonological complexity has been taken into account, the analyses by length and syllable complexity appear at the end of the "Preprocessing" subsection, right before the results. As for frequency, we have added the frequency of phonemes in the inventory in the subsection on the Tsimane' language and community at the end of the Introduction. We have also added analysis of performance as a function of phoneme and word shape frequency. Finally, in the results section, where it is relevant we focus on bisyllabic non-words, that are more easily compared with items used in previous work.

p.12 line 495 - what is similar about your study and [39]? This would be interesting to know and could shed light on what your results mean.

The items Tsegaye et al. (2011) used were very simple, simpler than items that were used in most of the other studies on adults. Therefore, we do not think Tsegaye's relatively lower results are due to their using complex non-words. We also notice that all papers on adults except for that one and Kosmidis were on Portuguese-speaking participants (most European Portuguese, de Loureiro's spoke Brazilian Portuguese). Tsegaye's participants spoke Amharic, whereas Kosmidis' spoke Greek (and they scored very highly). There is no salient similarity between our study and Tsegaye's. Of course, it is possible that differences in baseline performance across this published work reflects differences in frequency of child-directed speech across these cultures. We have not added this information in the text, since it is completely speculative.

p.13 line 540 I am not sure it is possible to compare this work with past developmental literature. Your work could be considered in light of what is out there, and can offer some contributions, but the ways the studies are conducted (necessarily very differently) means they are not in any way directly comparable.

We have modified the language in this section as follows: "Regarding the first strand of literature, we compared the NWR scores of Tsimane' children and adults against previous

developmental and adult literature. We are cautious in our interpretations because our study might differ from previous ones on many dimensions, and thus this is no perfect comparison."

p.13 line 544-553 It is not really possible to compare this work from work in literate cultures. At one point in this study there is a discussion about how the items in the Tsimane study may be harder than those used in previous work, but I don't think currently that the study can officially say these items are on par with previous work at all. The items probably are indeed harder conceptually for Tsimane people, as they are non-words, and non-words are strange for people, especially in a place where education is not common (and presumably language play is also not a known concept?).

These lines referred to a re-analysis of our results focusing on shorter items, and comparing them against scores found in previous work with shorter items. Since we added the sentence "We are cautious in our interpretations because our study might differ from previous ones on many dimensions, and thus this is no perfect comparison" just before this section, we feel readers have been warned about the general limitations of any comparison against published work. Regarding the specific point about non-words being "strange for people, especially in a place where education is not common (and presumably language play is also not a known concept?)", this is addressed in a new section in the Discussion ("Effects of input, literacy, or task oddity?"), where we raise the general point alluded to here: that the problem is that our task and/or the general non-word repetition idea might not be the perfect way to pick up on phonological processing.

p.13 line 560 - as it stands, it sounds a bit unethical to approach very young children who "systematically refused to participate". Was it simply that they didn't want to? Maybe this needs to be reworded.

This must be a misunderstanding - we meant that, in every group, the child who was youngest did not speak. We have removed the word systematically.

p.14 line 586 - there is so much research about the amount of talk children are exposed to, in different communities worldwide (using, e.g. Lena) - it is definitely important to review some of that here for more context.

We have not added citations to LENA work specifically because LENA does not distinguish between child- and other-directed speech, but we have rewritten this section as follows:

> ***"Another difficulty for this future enterprise is that the extent of stable individual variation in the amount of infant-directed speech among the Tsimane' is less known. In fact, although a few studies are coming out with direct measurements of child-directed speech estimates in more diverse populations [@weisleder2013talking;@cristia2017child;@casillas2019early], there are very few studies that are directly comparable, even when they seem to be using similar methods. We hold hope that cross-laboratory, cross-cultural collaborations like the ACLEW Project [@soderstrom2019quantifying] will be better able to fill this gap."***

p.14 line 591 onward - if the society is overall not very literate, then it seems almost impossible to separate the task effect (i.e. it is a strange task for them to participate in) from the "low" performance.

We have added a section to the Discussion ("Effects of input, literacy, or task oddity?"), where we raise the general point alluded to here: that the problem is that our task and/or the general non-word repetition idea might not be the perfect way to pick up on phonological processing.

p.14 line 610 - what did reference 36 find? More detail needed here.

The reference to a specific paper must have been confusing. We have rewritten the paragraph avoiding a reference to a specific paper so our point is clearer, as follows:

> ***"At present, we cannot distinguish effects of literacy from potential confounds. Some previous work comparing readers and non-readers made an effort to control for various confounds, for instance drawing participants from the same families to control for e.g. genetic predispositions and familial socio-economic status. In contrast, our present correlational design cannot control for such differences. In particular, the direction of causality may well be the opposite in our study: Adults who have developed short-hand-like phonological representations tend to learn how to read and stay longer in school than those with a different processing profile."***

p.14 line 615 - there has not been any real discussion of potential individual differences in the paper - I feel it would be useful here.

We decided not to dwell upon individual differences for this study, given the small N (and perhaps other reasons which the reviewer mentions). We feel that questions about individual differences are better addressed in follow-up studies with larger sample sizes and perhaps modifications to the study design (e.g. incentivized performance).

p.14 line 619 - if there are little differences between children and adults, then how do we really know that the experiment is getting accurate results? It is hard to be convinced about the task at this stage, because it really does seem like there is a "low reliability of the measure". If there really is clear evidence that the task works (such as the differences between lower vs better educated Tsimane people), then readers will need some more convincing about this point.

We believe this point relates to your general point: Are our findings due to input, literacy, or just our different way of administering the NWR task? We made no changes to this line, since that general comment is addressed by the new section in the Discussion ("Effects of input, literacy, or task oddity?". In that section we also make a convincing case that our task is indeed tapping phonological skills.

6. PLOS authors have the option to publish the peer review history of their article (what does this mean?). If published, this will include your full peer review and any attached files.

If you choose “no”, your identity will remain anonymous but your review may still be made public.

Do you want your identity to be public for this peer review? For information about this choice, including consent withdrawal, please see our Privacy Policy.

Reviewer #1: No

Reviewer #2: Yes: Dr. Debbie Loakes

[NOTE: If reviewer comments were submitted as an attachment file, they will be attached to this email and accessible via the submission site. Please log into your account, locate the manuscript record, and check for the action link "View Attachments". If this link does not appear, there are no attachment files to be viewed.]

While revising your submission, please upload your figure files to the Preflight Analysis and Conversion Engine (PACE) digital diagnostic tool, https://pacev2.apexcovantage.com/. PACE helps ensure that figures meet PLOS requirements. To use PACE, you must first register as a user. Registration is free. Then, login and navigate to the UPLOAD tab, where you will find detailed instructions on how to use the tool. If you encounter any issues or have any questions when using PACE, please email us at figures@plos.org. Please note that Supporting Information files do not need this step.
